# Local control: a hub-based model for the c-di-GMP network

**DOI:** 10.1128/msphere.00178-24

**Published:** 2024-04-09

**Authors:** Anna Vasenina, Yu Fu, George A. O'Toole, Peter J. Mucha

**Affiliations:** 1 Department of Mathematics, Dartmouth College, Hanover, New Hampshire, USA; 2 Department of Microbiology and Immunology, Geisel School of Medicine at Dartmouth, Hanover, New Hampshire, USA; The University of Iowa, Iowa City, Iowa, USA

**Keywords:** c-di-GMP, network, Lap system, *Pseudomonas fluorescens*

## Abstract

The genome of *Pseudomonas fluorescens* encodes >50 proteins predicted to play a role in bis-(3′-5′)-cyclic dimeric guanosine monophosphate (c-di-GMP)-mediated biofilm formation. We built a network representation of protein–protein interactions and extracted key information via multidimensional scaling (i.e., principal component analysis) of node centrality measures, which measure features of proteins in a network. Proteins of different domain types (diguanylate cyclase, dual domain, phosphodiesterase, PilZ) exhibit unique network behavior and can be accurately classified by their network centrality values (i.e., roles in the network). The predictive power of protein–protein interactions in biofilm formation indicates the possibility of localized pools of c-di-GMP. A regression model showed a statistically significant impact of protein–protein interactions on the extent of biofilm formation in various environments. These results highlight the importance of a localized c-di-GMP signaling, extend our understanding of signaling by this second messenger beyond the current “Bow-tie Model,” support a newly proposed “Hub Model,” and suggest future avenues of investigation.

## OPINION/HYPOTHESIS

Many bacteria utilize bis-(3′-5′)-cyclic dimeric guanosine monophosphate (c-di-GMP) signaling to regulate biofilm formation (1–3). One potentially powerful way to analyze a bacterium’s decision-making process to initiate biofilm formation is via a binary classification model (i.e., the organism is in one of two states), where input stimuli are processed to determine whether the organism stays in place (state 1 = biofilm) or leaves in search of more beneficial environments (state 2 = planktonic). Yan et al. (4), studying biofilm formation from this perspective, used the term “bow-tie signaling” to describe the shape of the c-di-GMP protein signaling architecture (4). The “Bow-tie Model” captures interesting aspects of the c-di-GMP network, grouping proteins into “makers” or “breakers” of this molecule and into receptors that respond to changing global pools of this second messenger. However, the model does not take into account additional information about, for example, the physical interactions between GGDEF domain containing c-di-GMP-synthesizing diguanylate cyclases (DGCs), HD-GYP/EAL domain containing c-di-GMP-degrading phosphodiesterases (PDEs), dual-domain proteins (with domains associated with both c-di-GMP synthesis and degradation) and/or c-di-GMP receptors, or the possibility of local pools of c-di-GMP participating in signaling.

Inspired by the Bow-tie Model, we investigated the ~50 proteins encoded in the *Pseudomonas fluorescens* Pf0-1 genome predicted to participate in the c-di-GMP signaling network and their several modes of second messenger regulation via the level of transcription, protein–protein interactions (PPI), and the extent of biofilm formation in response to environmental cues (5). Here, we used these published data in a multidimensional scaling analysis [principal component analysis (PCA) and statistical testing of features in logistic regression and random forest models] to better characterize the c-di-GMP signaling network of *P. fluorescens*. We found that measured growth of the wild-type strain in different environments, along with topological protein–protein interaction network features of the protein associated with a mutant strain, together demonstrated moderately successful prediction of strain phenotype (e.g., amount of biofilm formation) in a linear regression model. In contrast, our analysis suggests that expression of the genes coding for the c-di-GMP-metabolizing/-binding proteins in this system does not appear to make a significant contribution to the extent of biofilm formation across the many environments assayed. Thus, our data suggest a process driven by a network of protein–protein interactions is likely playing a key role in localized c-di-GMP signaling.

## RESULTS AND DISCUSSION

### Overview

An open question in the field of c-di-GMP signaling in bacteria is how organisms, with so many proteins dedicated to synthesizing, degrading, and binding this molecule, coordinate a coherent output for this signaling network, especially given that a second messenger like c-di-GMP is soluble and potentially freely diffusing. To begin to address this question, we first constructed mutations in all the known c-di-GMP-synthesizing, -degrading, and -binding proteins known at the time (6). Using these mutants, we determined the amount of biofilm formed by wild-type (WT) *P. fluorescens* Pf0-1 and each of these mutants in ~190 different growth conditions using Biolog assay plates as the source of the substrates (5). We also performed ~2,000 bacterial two-hybrid (B2H) assays in *Escherichia coli*, allowing us to determine the PPI network including almost 90% of the protein pairs in the c-di-GMP signaling network of this organism (5). Next, we assessed the expression of the genes coding for these proteins under 45 different growth conditions (5). Our original analysis, which was relatively simple (5), indicated a role of PPI and environmental signals as likely important inputs into the network, but provided no insight into the specific structure of the network nor did this analysis provide much insight into how to validate the signaling network.

To better understand how c-di-GMP regulatory proteins impact biofilm formation, we focused on the network representation of the B2H interaction network developed by testing ~90% of all possible PPI among the DGCs, PDEs, dual-domain proteins, and c-di-GMP receptors of *P. fluorescens*. For this analysis, the nodes of the PPI network are the proteins, and the edges between the proteins represent their ability to interact with each other according to the results of the B2H experiment. There are three important questions we wanted to address. First, what do various node centrality measures (that is, metrics of the importance of each node within a network) tell us about the specific role of each protein and the structure of this molecular network as a whole? Second, how does the PPI network relate to the extent of biofilm formation in response to environmental input cues and to the level of transcription of genes coding for the c-d-GMP-metabolizing/-binding proteins? Finally, what information is most valuable in predicting biofilm phenotypes? We address each of these points below.

### Probing the topology of the PPI network

**Fig 1:**
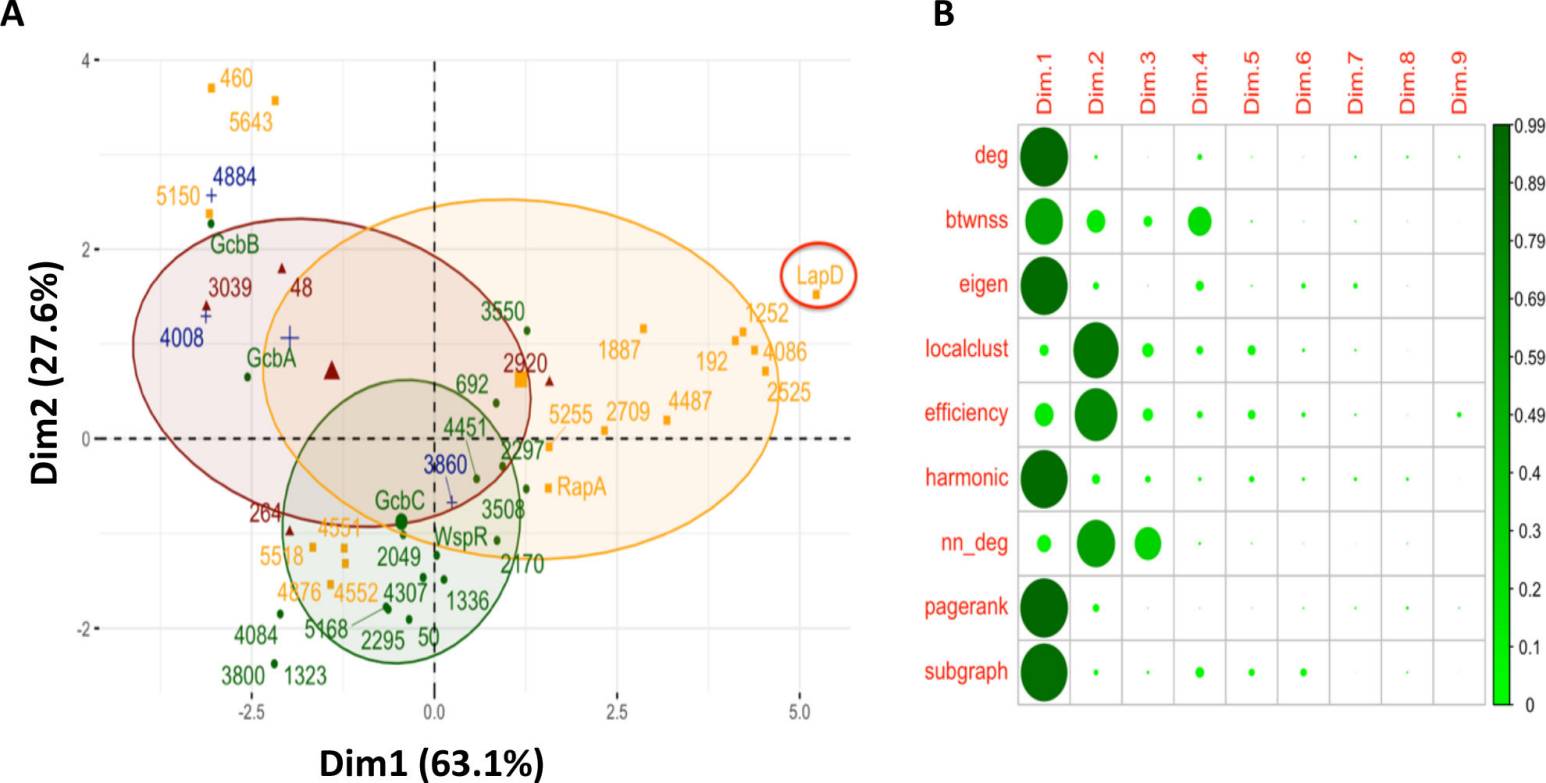
Interrogating the PPI network. (A) PCA on node centralities in the largest connected component of the PPI network. Colors indicate domains: yellow indicates proteins with dual domain architecture, green indicates DGCs, red indicates PDEs, and blue indicates c-di-GMP-binding PilZ domains. The numbers indicate gene number assignments from the *P. fluorescens* Pf0-1 genome, while names are used for previously reported proteins (6). LapD is a previously reported c-di-GMP receptor critical for the cell surface localization of the key biofilm adhesin LapA (1, 7). Note the clusters of dual domain proteins that map away from the cluster of proteins near LapD (far right) in the lower left quadrant (Pfl_4552, Pfl_4554, Pfl_4876, and Pfl_5518) and the upper right quadrant (Pfl_0460, Pfl_5643, and Pfl_5150). These two clusters are separated in the second principal dimension, which has lower explanatory power. These proteins also represent low-degree interactors of degrees 1 through 6 (see Fig. 2) and may represent dual-domain proteins that largely function outside the connected network (see text for additional details). (B) Feature loadings of each centrality (left labels) on each PC dimension (top labels). Larger and darker circles indicate that a larger percentage of the centrality feature aligns with that PC.

First, to gain insight into the topology of this PPI network, we computed nine node centrality measures that characterize each protein’s interaction network (Table 1) and then performed PCA (Fig. 1A) on these nine features.

**TABLE 1:** The description of the nine network centralities used to measure the interaction behavior of each protein in the c-di-GMP signaling network

Network centrality	Description	What we learn
Degree	The number of interactors a protein has with other members of the network	How connected you are
Betweenness	How often the protein acts as a bridge between signaling clusters	How you connect groups
Local clustering coefficient	Measures how often proteins that share an interactor interact among themselves	Are your friends, friends with each other
Eigenvector centrality	Computes node importance accounting for all indirect connections (via an adjacency matrix)	The extent of your network
PageRank centrality	Measures node importance by recursively trickling information from one node to another	Where the signal is likely to end up
Harmonic centrality	How many steps from a given protein to all the rest of the proteins in the network, on average	Connectivity across the network (Kevin Bacon effect)
Local efficiency	Computes how well connected the neighborhood of interactors is without the protein of interest	Connectivity across the network, leaving out the hub protein
Subgraph centrality	Measures the number of network motifs a protein participates in, weighting them according to their size	Number of potential pathways a protein has, weighted according to their length
Average nearest neighbor degree	Computes the average interaction degree of proteins with which the node interacts	How connected are your neighbors

**Fig 2:**
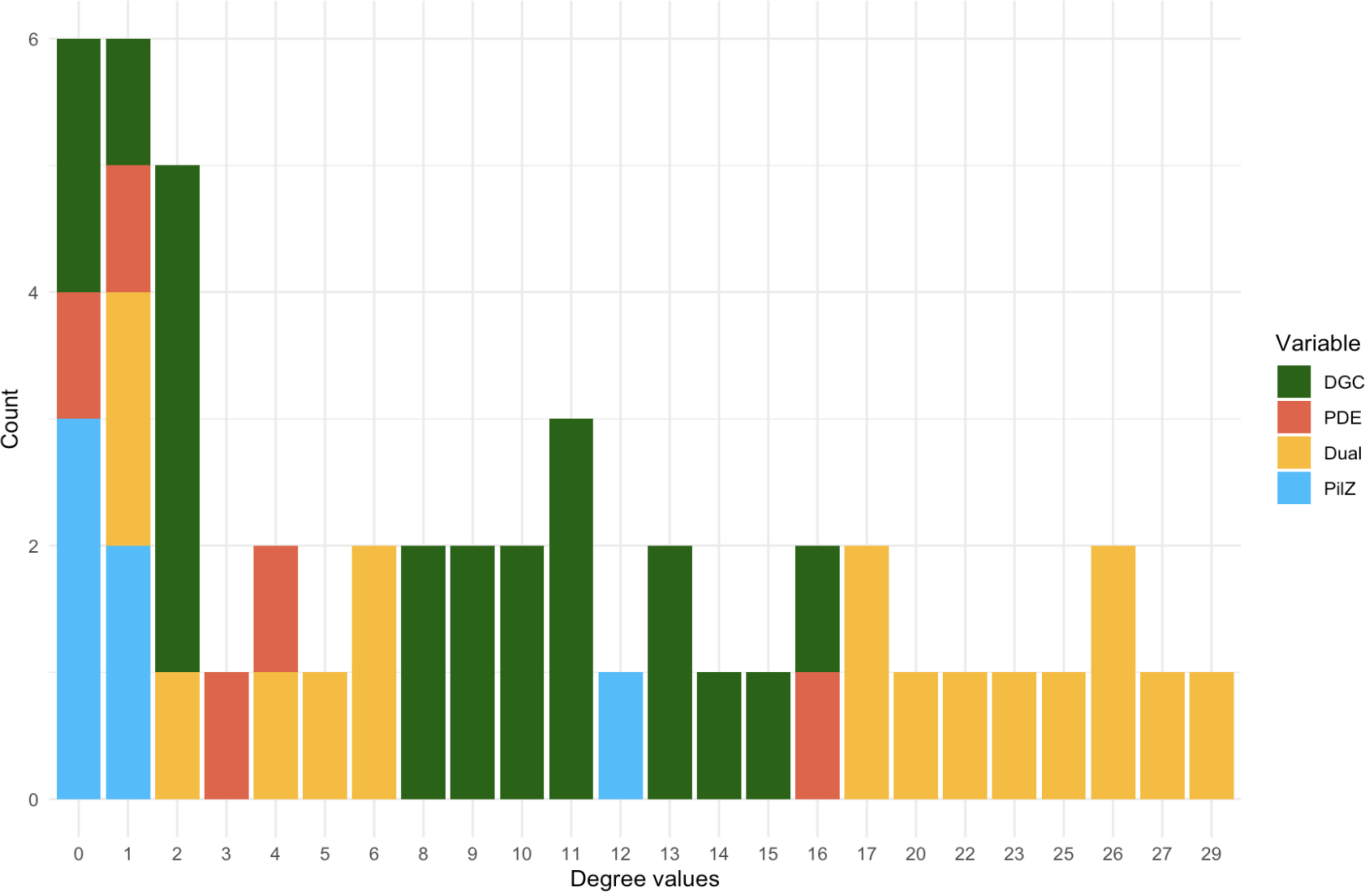
Degree values of proteins in the PPI network, colored by domain. All nodes in the PPI network of degree 17 and higher are dual-domain proteins. Nodes of degree values between 8 and 16 are primarily DGCs, while most proteins with PilZ domain have either degree 0 or 1. PDEs mostly take on degree values between 0 and 4. LapD has a degree of 29. The degree values are discussed in more detail in the text.

We found that most of the dual-domain proteins, which contain both GGDEF and EAL domains, tend to have high scores along the first principal component, which is mostly explained by degree and PageRank centralities (Fig. 1A and B). The degree and PageRank scores (8) imply that dual-domain proteins serve as regulatory hubs, as they receive inputs from many other proteins (for a more in-depth description of network centralities measures, see Table 1 and the supplemental material). Consistent with this conclusion, the membrane-bound, c-di-GMP receptor LapD has the highest score along the first principal component (Fig. 1A, far right, red circle). These proteins are also defined by their extensive number of PPI with other members of the network, spanning from a minimum of 17 for two of the dual-domain proteins to a high of 29 interactions for LapD (Fig. 2). This finding is consistent with signals converging on LapD, a key regulator of the level of a critical cell-surface, biofilm adhesin LapA. Cell-surface LapA ultimately governs biofilm formation by *P. fluorescens* (1, 7).

Notice two clusters of dual-domain proteins that do not map near LapD (Fig. 1A). The first such cluster, comprised of three proteins (Pfl_0460, Pfl_5150, and Pfl_5643) lies in the upper left quadrant of the PCA plot. These proteins display one or two interactions with other proteins in the network (compared to 29 for LapD). We posit that Pfl_0460, Pfl_5643, and Pfl_5150 may be proteins participating in the “Bow-tie” aspect of c-di-GMP signaling, which does not require any protein–protein interactions to access the pool of cellular c-di-GMP (4). The second cluster of dual-domain proteins mapping away from LapD is comprised of the proteins Pfl_4551, Pfl_4552, Pfl_4876, and Pfl_5518, which display an intermediate level of interaction with proteins in the network (between four and six interactions, Fig. 2), and interestingly lie among DGCs in the lower left quadrant in the PCA plot (Fig. 1A). Based on their clustering, we would predict that the dual-domain proteins Pfl_4551, Pfl_4552, Pfl_4876, and Pfl_5518 may very well be dominated by their DGC activity and/or participate in DGC-centered signaling. The sequences of Pfl_4551 and Pfl_4876 indicate that they are indeed active DGCs, while Pfl_4552 and Pfl_5518 likely have PDE activity. Interestingly, the DGC Pfl_4551 and PDE Pfl_4552 are adjacent genes on the genome, which is often associated with their participation in similar functions.

In contrast to the dual-domain proteins, DGCs exhibited high measures on the second principal component, which is best described by local clustering and average nearest neighbor degree (8) (Fig. 1A and B). Most DGCs interact with between 8 and 16 other proteins in the network, below the degree of interaction of most dual-domain proteins (Fig. 2). That is, DGCs are more dominated by local interactions versus dual-domain proteins, the latter of which display a more extensive series of interactions across the network. Since the neighbors of DGCs include c-di-GMP-binding proteins (i.e., the PilZ-domain protein Pfl_3860 clusters with the DGCs on the PCA plot, Fig. 1A), this result confirms a local dynamic wherein c-di-GMP produced by these DGCs is delivered to a spatially proximal c-di-GMP-binding protein. For *P. fluorescens*, there is at least one well-documented example of the requirement of local signaling driving the c-di-GMP network response (9). Interestingly, multiple PilZ proteins show a low degree of interaction, (i.e., Pfl_4008 and Pfl_4884 are found in the upper left of the PCA plot, Fig. 1 and 2)—we would predict that these proteins are also likely participating in Bow-tie, rather than localized, signaling.

Therefore, Fig. 1 shows that proteins of different types have a predictable PPI network behavior that allows us to distinguish them based on their node centrality measures. To further probe these relationships, we tried to classify DGCs, PDEs, and dual-domain proteins based solely on their network features. The combination of features that produced the highest area under the receiver-operating characteristic (ROC) test scores for each protein type (DGC, PDE, and dual domain) in logistic regression models included eigenvector centrality, betweenness, and PageRank (see Table 1). Here, the ROC curve is formed by plotting the true-positive rate (sensitivity) against the false-positive rate (specificity) at various decision thresholds. Thus, the area under the curve (AUC) derived from the ROC plots represents the probability that the model correctly distinguishes between positive and negative classification instances, where a score of 0.5 indicates that the model’s predictive ability is that of random guessing, while a score closer to 1 indicates a better discrimination ability. The three eigenvector centrality, betweenness, and PageRank features together give an AUC score of 0.925 (with bootstrapped median AUC over 100 runs of 0.923, with empirical 95% CI of 0.840–0.988) for binary classification of whether the protein is dual domain (or not), 0.898 (median 0.919, CI 0.826–0.993) for DGCs, and 0.743 (median 0.760, CI 0.636–0.953) for PDEs (Tables S1 and S2). Thus, these network features accurately predicted whether a protein was a DGC or dual-domain protein >90% of the time and a PDE ~75% of the time.

We also classified protein types using twofold cross-validated random forest models. The degree network feature alone, which measures the number of interactors a protein has with other members of the network, predicts DGCs, PDEs, and dual-domain proteins with a 0.80 average test accuracy (Fig. S1). That is, even if we give only half of the true labels to a random forest model, it can classify the other half quite accurately. This is not surprising once we consider the PPI network degree distribution in Fig. 2, where we see that the domain types mostly fall neatly into specific ranges of the degree distribution, making them easy to classify. Together, these data indicate that *network behavior* (i.e., protein–protein interaction features) allows us to accurately predict whether a c-di-GMP signaling component is a DGC, PDE, or dual-domain protein.

With these classification results, we confirmed that not only do different c-di-GMP-related protein types (DGC, PDE, and dual domain) exhibit different behaviors in the PPI network of this organism (as observed in the PCA, Fig. 1 and 2), but these proteins are also readily distinguishable in prediction models by those behaviors (described by the network features). While these results might not hold for proteins of a different c-di-GMP signaling network, we believe that exploration of the PPI networks in other bacteria can also prove insightful for investigation of c-di-GMP-mediated regulatory mechanisms of biofilm formation.

### Analysis of a combination of signaling mechanisms

With the above promising results from the PPI network analysis, we sought to delve deeper into the combinations of additional data sets we reported previously (5). We attempted to build a model for predicting the extent of biofilm formation (i.e., biofilm biomass) that incorporates information from all three of our available data sets. That is, in addition to the PPI network described above, we had previously examined the amount of biofilm formed by mutant strains lacking individual components of the c-di-GMP network in different environments, as well as the level of transcription of genes coding for these c-di-GMP-metabolizing/-binding proteins under 45 different growth conditions (5).

**Fig 3:**
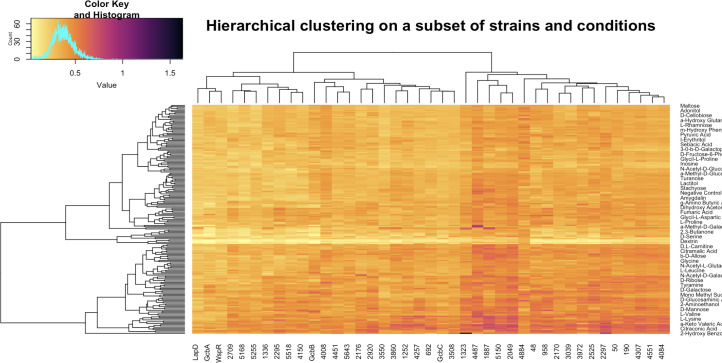
Hierarchical clustering of strains (columns) and carbon-source environments (rows) according to measured biofilm production. Biofilm production values have been batch normalized according to wild-type measurements in the corresponding batch. Harmful medium components, such as detergents, and the eight mutants, all lacking c-di-GMP degrading PDEs and forming the most robust biofilms across all conditions (see Fig. S2), have been removed prior to clustering. If environments had been the only defining factor in the amount of biofilm formed, then the clustering would show perfect row ordering of environments by the amount of biofilm formed from lowest to highest, on average, across all strains. Conversely, if only strains regulated biofilm formation, only the columns would have been permuted to create such ordering. In the present case, the algorithm permutes both strains and environments, indicating that both play a significant role in regulating biofilm formation.

First, we leveraged the one-to-one correspondence between interacting proteins and strains that lack the cognate gene. Dahlstrom et al. grew 49 mutant strains, plus the WT as a control, in 188 environments using Biolog PM1 and PM2A nutrient plates (which include a large range of possible carbon sources), then recorded the amount of biofilm formed by every strain in each of the environments using the crystal violet-based biofilm assay in 96-well dishes (5). We visualized these data using hierarchical clustering (Fig. S2). The amount of biofilm formed varies widely depending on the strain and the environment. First, we note that eight mutant strains (with the following genes deleted: Pfl_0460, Pfl_4086, Pfl_264, Pfl_4876, Pfl_0192, *rapA*, Pfl_4552, and Pfl_3800), which are candidate or demonstrated PDEs, showed robust biofilm formation in all growth conditions, suggesting that they were producing high levels of c-di-GMP independent of the environmental inputs. This finding of robust biofilm formation across environments in strains lacking a PDE has been reported previously (10, 11). Furthermore, we observed that for some of the environmental conditions, the biofilm production was always low; these wells of the Biolog plates contained detergents, which inhibited growth of the bacteria (5). After removing the eight constitutively hyper-biofilm-forming strains and the detergent-containing medium conditions from the analysis, the visualization of the hierarchical clustering heatmap in Fig. 3 continues to show strong differences between strains (the different columns in the figure). After considering negative controls and average WT growth in each environment, we found that the variation across strains makes a comparable impact on biofilm formation to the impact of the environment in which the mutant is grown, as the median standard deviation of strains across environments is 0.0932 [interquartile range (IQR) 0.0845–0.1017], while the median standard deviation of environments across strains is 0.0769 (IQR 0.0657–0.0880). That is, the gene in the c-di-GMP network and the environment (i.e., carbon/energy source provided in the biofilm assays) contribute to a similar extent to the resulting biofilm phenotype.

The last feature we attempted to incorporate into our c-di-GMP signaling prediction framework is gene expression. In a previous study, we examined the expression of the genes in the c-di-GMP network in 45 different carbon/energy sources (5). We excluded *rapA* as an outlier, as it was shown previously to have a large change in transcription in response to a low-phosphate environment (12). As was done in Fig. 3 for biofilm production, we hierarchically clustered expression measurements for each gene across environments, obtaining a very narrow standard deviation distribution of gene expression levels (as measured via z-scores), indicating lack of gene expression changes for most genes in most growth conditions (Fig. S3). Additional investigation into up- or downregulation of transcriptional levels in different environments for interacting proteins showed no statistical significance in linear regression models (Fig. S4 and S5; Table S3). That is, there was no evidence that the genes coding for interacting proteins displayed coordinated gene expression patterns.

### Building phenotype prediction models

Finally, we attempted to build a phenotype prediction model. We excluded transcription data due to the low variance in gene expression (i.e., low information content) across the multiple environments and genes tested and the lack of statistical significance in our preliminary models. That is, when we add gene expression levels as a feature to our regression models, the adjusted R-squared stays the same or drops due to penalization (i.e., adjusting for additional variables with no increase in information).

To build models, we took advantage of key aspects of our previously reported data set (5). Since every mutated strain results in a single protein being removed from the network and we have a corresponding set of data from the PPI analysis, we can use the PPI network measurements of these proteins as features in phenotype linear regression models. We predicted that the PPI data might help differentiate strains in some environments where the nutrients added to the medium do not dictate the biofilm formed by a majority of strains. Thus, we included in the model the amount of biofilm biomass formed in each environment by the wild-type strain, along with PageRank, betweenness, and eigenvector centrality values of the PPI network, since this combination of network features predicted the domain architecture of a protein most accurately in a logistic regression model (see Tables S1 and S2 and text above). Additionally, we were curious how a simple binary indicator of whether a protein is a dual-domain type contributes to phenotype prediction, and thus, this variable was also included in our regression model. While network features above serve as a proxy for protein domain type, they also incorporate additional information about protein interactions.

The adjusted R-squared of the model described in Table 2 is 0.4391. We found it promising that all independent variables included in this model were statistically significant. Nevertheless, given the relatively large number of inputs in the model (49 strains tested across 188 environments contribute to ~9,500 biofilm biomass inputs the model could fit), we rigorously tested for overfitting using permutation tests. Overfitting often occurs when the number of observations and, hence, degrees of freedom, is so large (as in our case) that the model in essence learns noise from the training data rather than a true underlying pattern, leading to poor generalizations on subsequently analyzed test data. Thus, we employed a series of random shuffles on the whole input data set, as well as exclusively on PPI data, to generate a randomized null distribution to assess whether the model’s performance is significantly better than expected by chance. Figure S6 shows that the model did not overfit to the independent variables, further supporting the statistical significance. Moreover, we note that the simplest linear model, with biofilm production specified by the amount of biomass produced by the wild type, has an R-squared of 0.376. This baseline R-squared value indicates that the four PPI network centrality variables (PageRank, betweenness, eigenvector centralities, and dual-domain binary indicator variable), taken together, provide additional explanatory power increasing R-squared by 0.06. Nevertheless, permutation testing of only the PPI network centralities effectively eliminated this additional contribution in all permuted models, confirming that the protein information about the strains, i.e., the explanatory power that the protein variables bring to the regression model is strongly statistically significant.

**TABLE 2:** Linear model coefficients and *P* values of independent variables across 49 strains and 192 environments used to test for the biofilm biomass formed

	Coefficient	*P* value^*a*^
(*Intercept*)	0.0091231	0.0825 (*)
*Wild type*	1.0312640	<2e−16 (****)
*Eigenvector centrality*	−0.1353696	2.65e−09 (****)
*Betweenness*	−0.0008525	<2e−16 (****)
*PageRank*	3.0825489	7.05e−07 (****)
*Dual-type indicator*	0.0720563	<2e−16 (****)

^*a*^ All variables apart from the intercept show very high statistical significance (****P < 0.0001 and *P < 0.1). We suspect that the wild-type variable (amount of biofilm), which provides the only information about the environment in this model, primarily serves as an intercept given that its coefficient is ~1.

Thus, the adjusted R-squared contributions from all variables are statistically significant, and our model is robust. From this analysis, we conclude that the physical interactions between DGCs, dual-domain proteins, and PDEs, contribute to a statistically significant mode of regulation of biofilm formation. Signaling via these PPI is further modulated by the nutritional cues present in the environment, which play a large and statistically significant role in our regression model as well. However, none of the models we considered identified any statistically significant transcriptional control impacting the biofilm formation.

### Conclusions

**Fig 4:**
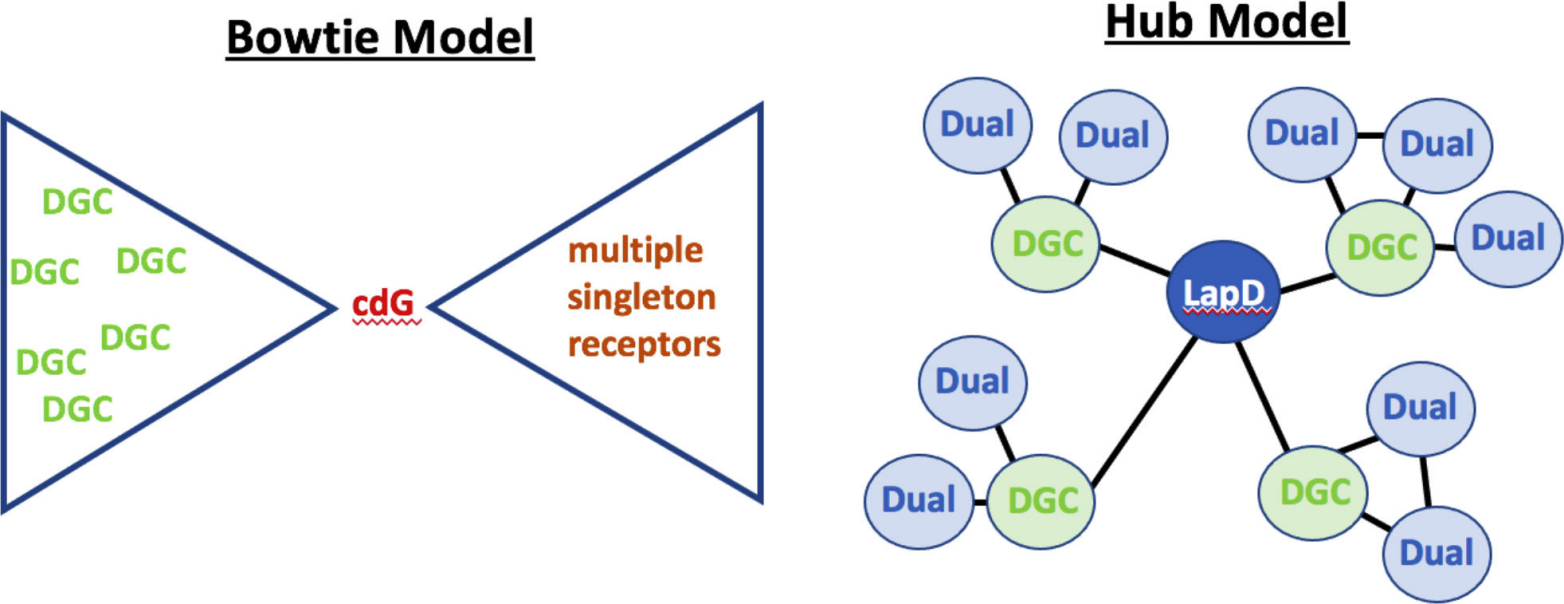
Models of c-di-GMP signaling. On the left is shown the previously reported “Bow-tie Model,” which posits the production of a pool of c-di-GMP from a series of DGCs, which in turn is detected by multiple singleton receptors. This model invokes a global pool of this second messenger. On the right is shown our “Hub Model,” which posits a role for localized signaling driven by a series of PPI interactions. The extent of these interactions can be used to accurately infer the class of enzyme (i.e., DGC versus dual-domain protein). An induced subgraph of the actual PPI network with eight highest-degree dual-domain proteins and DGCs that interact with them is shown in Fig. S8 to support the “Hub Model” visualization.

Overall, our analysis here provides a framework for exploring an alternative to the Bow-tie Model (Fig. 4, left), which we refer to as the “Hub Model” (Fig. 4, right), which accounts, at least in part, for the increasing examples of localized signaling (4). Our analysis leads us to the conclusion that node centrality measures of each protein, that is, aspects of interactions in the PPI network, can serve as meaningful features in regression models that attempt to predict biofilm phenotypes. Moreover, the PPI network behavior of each protein type (DGC, dual-domain, PDE, and PilZ) is unique, and we can accurately classify the domain type of any protein by assessing their network centrality values. Thus, from this analysis we conclude that the different proteins in the c-di-GMP network (i.e., DGC, dual-domain, PDE, and PilZ) likely have distinct and characteristic roles to play in the network.

The statistical robustness of our regression biofilm phenotype model and classification models asserts the notable, and rigorously statistically tested, contribution of the PPI network to the regulation of biofilm formation. Although the specific findings here might not be directly transferable to proteins of another c-di-GMP signaling system, we suggest that investigating the PPI networks in other bacterial species might yield valuable additional insights into the regulatory mechanisms governing c-di-GMP-mediated biofilm formation. While we encourage the analysis of PPI network of c-di-GMP regulatory proteins and the exploration of network significance in understanding the biofilm formation phenotype in other microbes, we note that larger-scale predictions across multiple environments for multiple genotypes are vulnerable to some random effects of protein-independent variables, as evidenced by controlled permutation testing (Fig. S7). More sophisticated regression models could be employed in future analyses but must be carefully examined for any random effects.

It is important to note some of the caveats of this analysis. First, we emphasize that the Hub Model and previously reported Bow-tie Model (4) are unlikely to explain *all* aspects of c-di-GMP signaling. Rather, we propose the Hub Model both to expand on the currently reported signaling mechanisms and to provide a framework going forward for experiments to validate this model. For example, how do the interactions between multiple DGCs with LapD (see Fig. 4, right) control biofilm formation? Is there a preferential hierarchy of interactions, or do PPI interactions respond to environmental inputs? The latter is a real possibility given that LapD-interacting DGCs have ligand-binding CACHE domains (5, 6) that are likely to respond to environmental inputs. Indeed, our regression models indicate that combining information from the PPI data and nutritional cues provides the greatest predictive power of biofilm biomass formed, while keeping the model statistically robust, suggesting a mechanistic connection between these two nodes of regulation. For *P. fluorescens*, there is an example of local c-di-GMP-mediated out modulated by an environmental cue (9), perhaps indicating a general mechanism of signaling. Finally, while our data set is rich, it lacks some key information known to drive c-di-GMP signaling, including post-transcriptional regulatory features [i.e., regulation by the CsrA/RsmA signaling pathway (13–16)], a role for other catalytically inactive GGDEF/EAL domain proteins that may act as receptors, yet-to-be-identified c-di-GMP-binding proteins, as well as interactions of c-di-GMP-binding/-metabolizing proteins outside the core second messenger signaling system (17–20).

Finally, while this c-di-GMP signaling network is relatively large for such systems, compared to the data sets usually probed with the network analyses used here, the relatively small size of the data set and lack of large variation in PPI network behavior mean that the node centralities employed here (Table 1) provide only an initial view into the actual protein regulatory mechanisms. Moreover, the PPI network determined using the heterologous B2H system in *E. coli* captures only potential interactions, but it does not provide any information about whether those interactions occur in the native organism. Given these caveats, we find the statistical significance of PPI determined in *E. coli* and their explanatory power for the biofilm phenotype in *P. fluorescens* remarkable. Furthermore, the data presented here expands beyond previous models of “local control,” which were not defined sufficiently at a system level to generate testable hypotheses. In contrast, our analysis is novel in that it paints a more precise picture of how PPI plus nutrient inputs could modulate c-di-GMP signaling distinct from a global second messenger pool (Fig. 4, right), thereby providing a framework that can be validated experimentally going forward.

## SUPPLEMENTAL MATERIAL

Supplemental Figures msphere.00178-24-s0001.pdf Figures S1 to S8.

Supplemental Text msphere.00178-24-s0002.pdf Supplemental methods.

Supplemental Tables msphere.00178-24-s0003.pdf Tables S1 to S3.

ASM does not own the copyrights to Supplemental Material that may be linked to, or accessed through, an article. The authors have granted ASM a non-exclusive, world-wide license to publish the Supplemental Material files. Please contact the corresponding author directly for reuse.
