# Supplementary material for: Local control: a hub-based model for the c-di-GMP network
Source: mSphere. 2024 Apr 9;9(5):e00178-24. doi: 10.1128/msphere.00178-24 (PMC11237430; doi:10.1128/msphere.00178-24)
Supplement: Supplemental Figures — Figures S1 to S8. [file msphere.00178-24-s0001.pdf]

SUPPLEMENTAL FIGURES

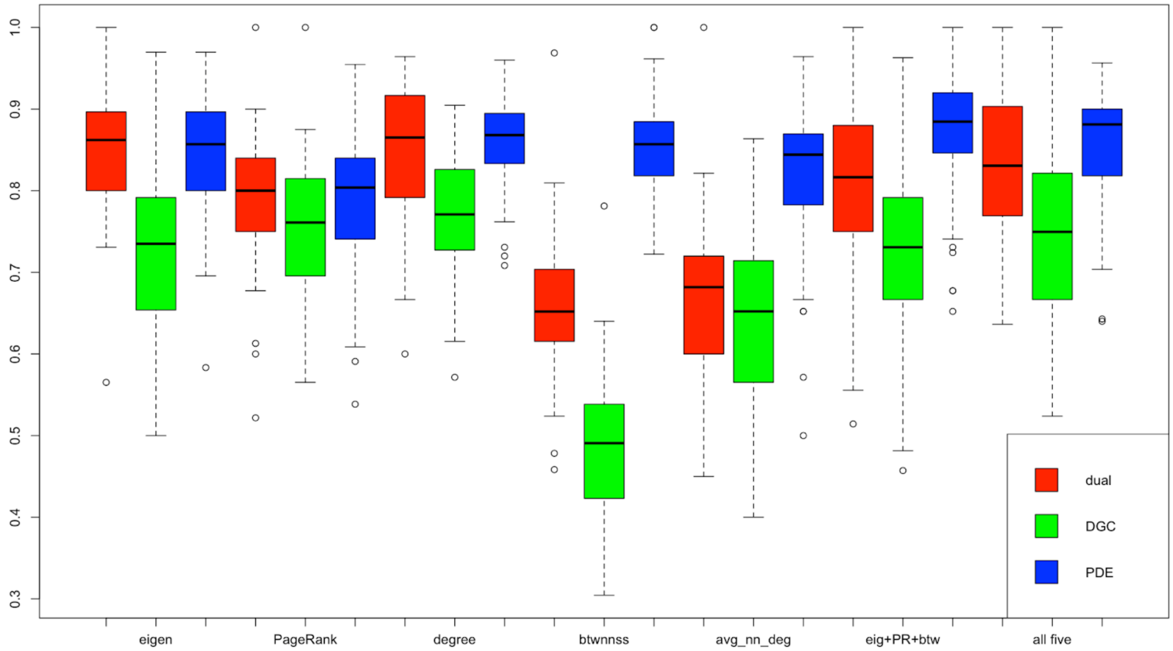

Figure S1: Accuracy boxplots for seven random forest models for each protein type. We performed 50 random forest analyses on three protein type indicator variables (dual domain, DGC, and PDE) with randomized 50/50 train-test split for seven combinations of network features in the order from left to right above: eigenvector centrality, PageRank, degree, betweenness, average nearest neighbor degree, eigenvector + PageRank + betweenness, and all five features combined. Each boxplot contains accuracy scores of one random forest model for one of the indicator variables colored by domain type: red indicates dual domain, green indicates DGC, and blue indicates PDE.

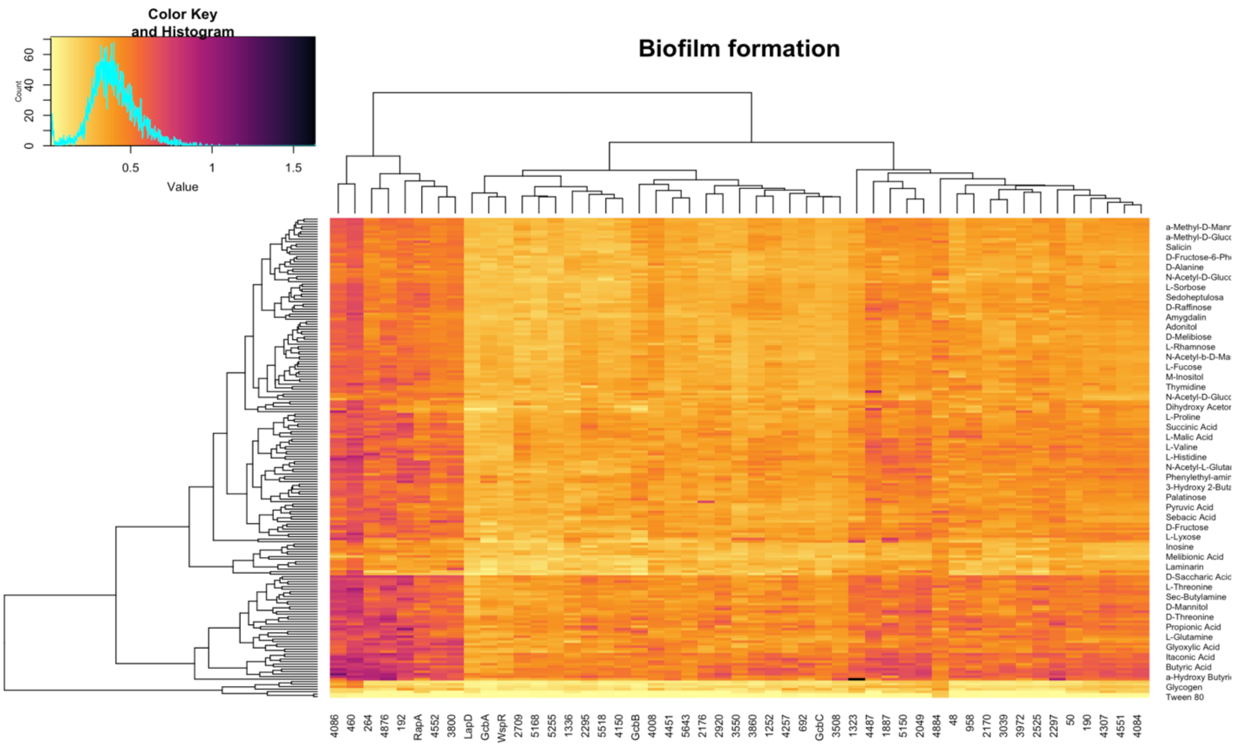

Figure S2: Heatmap of hierarchical clustering of biofilm formed on all strains and environments, normalized by WT batch median. As in **Figure 3**, hierarchical clustering is performed on the distance matrix of the raw data with reordering of rows and columns. Column clustering seems to dominate row clustering, grouping phenotypes mostly by strains that have a larger influence on the amount of biofilm formed. To control potential skewing of the z-score values due to known high-formation strains and detergents that kill all bacteria, we removed the detergents (Tween) and the strains lacking the genes Pfl_0460, Pfl_4086, Pfl_264, Pfl_4876, Pfl_0192, *rapA*, Pfl_4552, Pfl_3800 (leftmost columns). Loss of these genes is known to promote robust biofilm formation across every environment tested. The hierarchical clustering of the modified dataset with omitted rows and columns is shown in **Figure 2**.

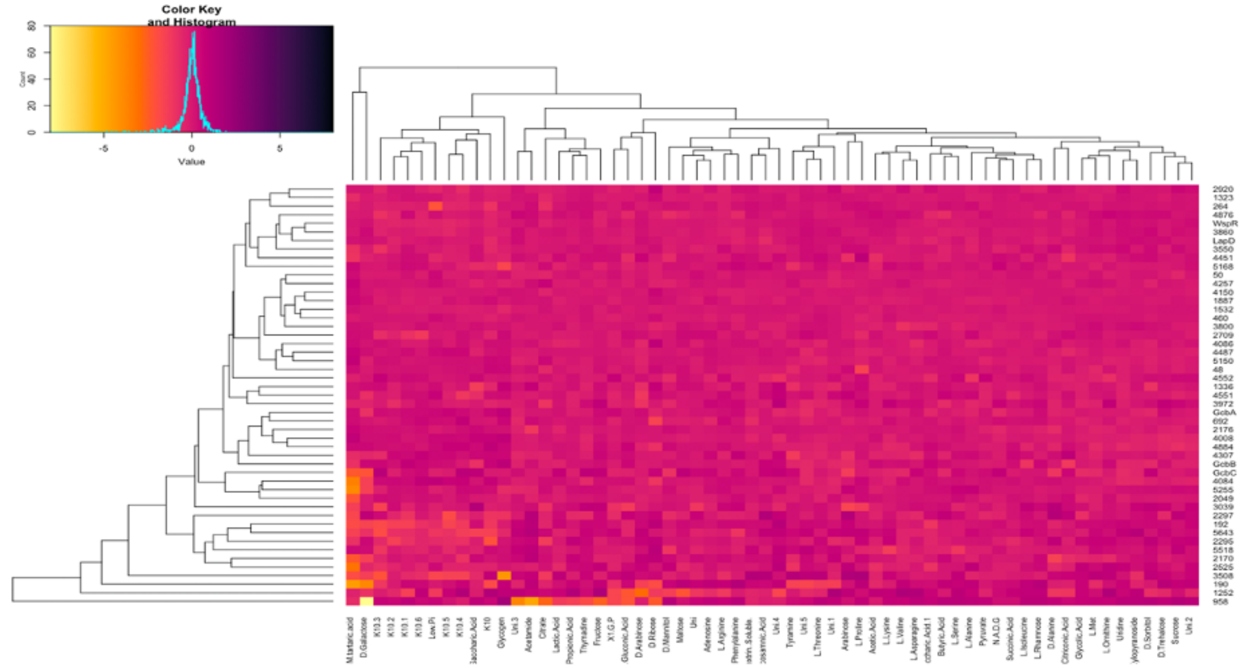

Figure S3: Heatmap of hierarchical clustering of z-scores of transcription levels by environment using (columns) in each strain lacking the respective gene (rows). M-tartaric acid, D-Galactose and K10 medium induce more variation of transcription levels and tend to down-regulate several genes, as evidenced in the left lower corner. The genes Pfl0_958 and Pfl0_1252 are down-regulated in several different environments, while the expression of gene Pfl0_0190 is significantly reduced in glycogen medium.

A **B**

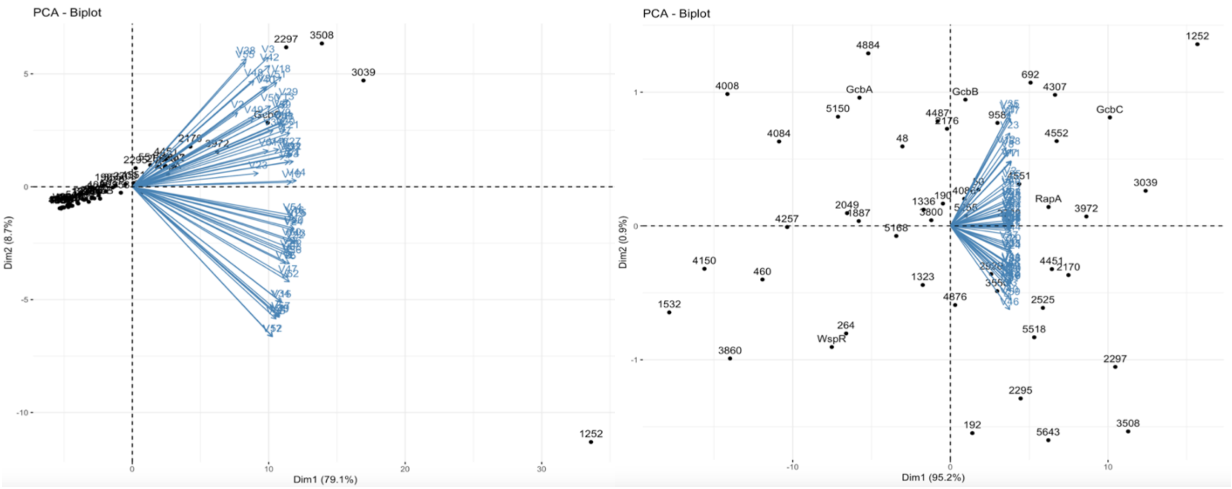

Figure S4: **PCA of gene expression with respect to the *lapD* gene in 192 environments**. After we computed the ratio of all genes with respect to *lapD* in each environment, we performed PCA analysis on all environments before and after we calculated the logarithm of the ratios. We performed the same PCA analysis of additive log ratios for all reference genes and discovered that their biplots looked similar to the ones shown here. We believe this supports the lack of variation on the z-score heatmap. In the left panel we considered transcription level ratio of all genes to *lapD*, which indicate that in almost all environments the genes do not change their transcription levels drastically compared to the *lapD* gene. The logarithm of those ratios smooths out any outliers with the first principal component explaining 95.2% of variation (right panel). We chose the *lapD* gene as a reference gene due to its importance in the signaling mechanism, however, other reference genes produce similar results with no significant outliers in the PCA of log ratios (not shown).

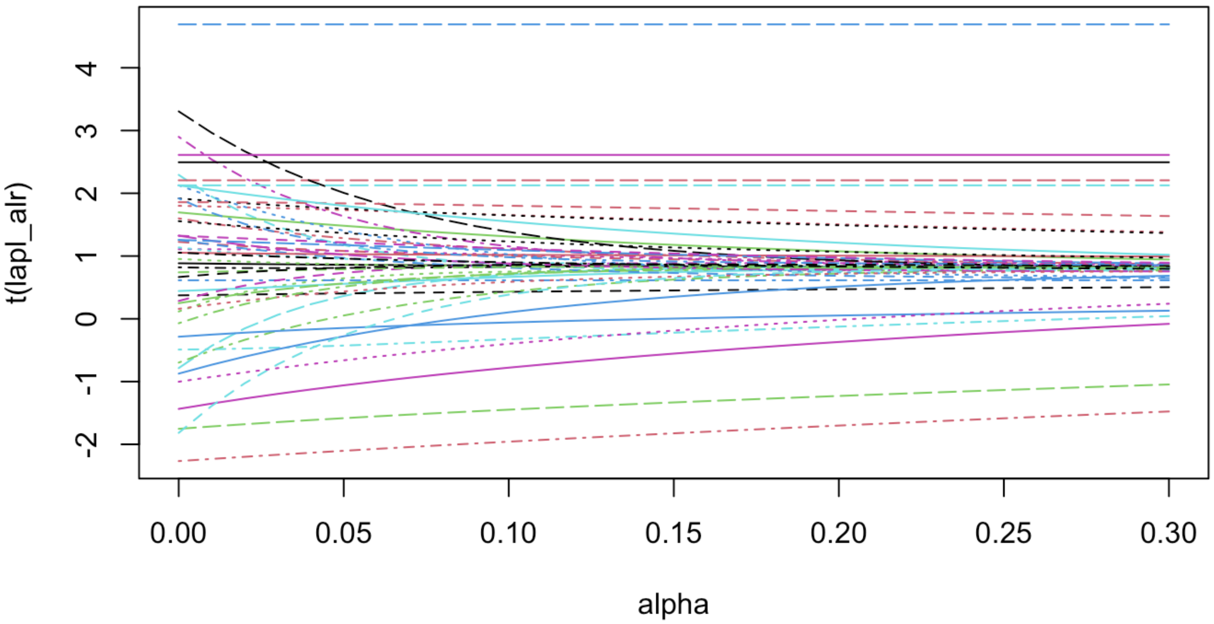

Figure S5: **Exponential diffusion of gene expression data for parameter alpha.** One might reasonably wonder if there is a synchronous up- or down-regulation of transcriptional levels in different environments for interacting proteins. That is, do proteins that physically interact show coordinated changes in gene expression? To investigate this possibility, we processed the unscaled expression data as follows: first, accounting for compositional nature of the expression data, as it is conventionally normalized per 1000 reads, we converted the values into additive log ratios, choosing *lapD* as a reference gene, given its importance as the key receptor. To incorporate expression data into network topology we gradually diffused the processed transcription levels on the network with the exponential of the graph Laplacian. Therefore, the feature input of gene *i* in environment *j* contains information about smoothed-transcription levels of all nodes in the network in the environment *j* with respect to node *i.* For instance, the neighbors of node *i* would have a larger impact on the value at *i* as opposed to the genes located further away in the network. The diffusion of transcription data on the network was built using the standard graph Laplacian *L=D-A*. The log ratios with respect to *lapD* were diffused by the matrix exponential $e^{-\alpha L}$ at $\alpha = 0.025$, where the level of diffusion seemed sufficient but hasn't yet reached an equilibrium. This smoothed transcription information propagated through the entire network did not show any significance in phenotype prediction models (**Table S3**). Shown here are the transcription level ratios with respect to the *lapD* gene in each environment diffused by the matrix exponential for different values of the diffusion parameter alpha. Each gene ratio is indicated by a line. At alpha = 0.00, the gene data is not diffused, giving the original values of transcription level ratios on the y-axis, and at alpha = 0.30, the diffusion almost reaches equilibrium with most gene data smoothed out across the PPI network. For linear regression model we chose the diffused gene data at alpha value of 0.025 for moderate spread of information on the network.

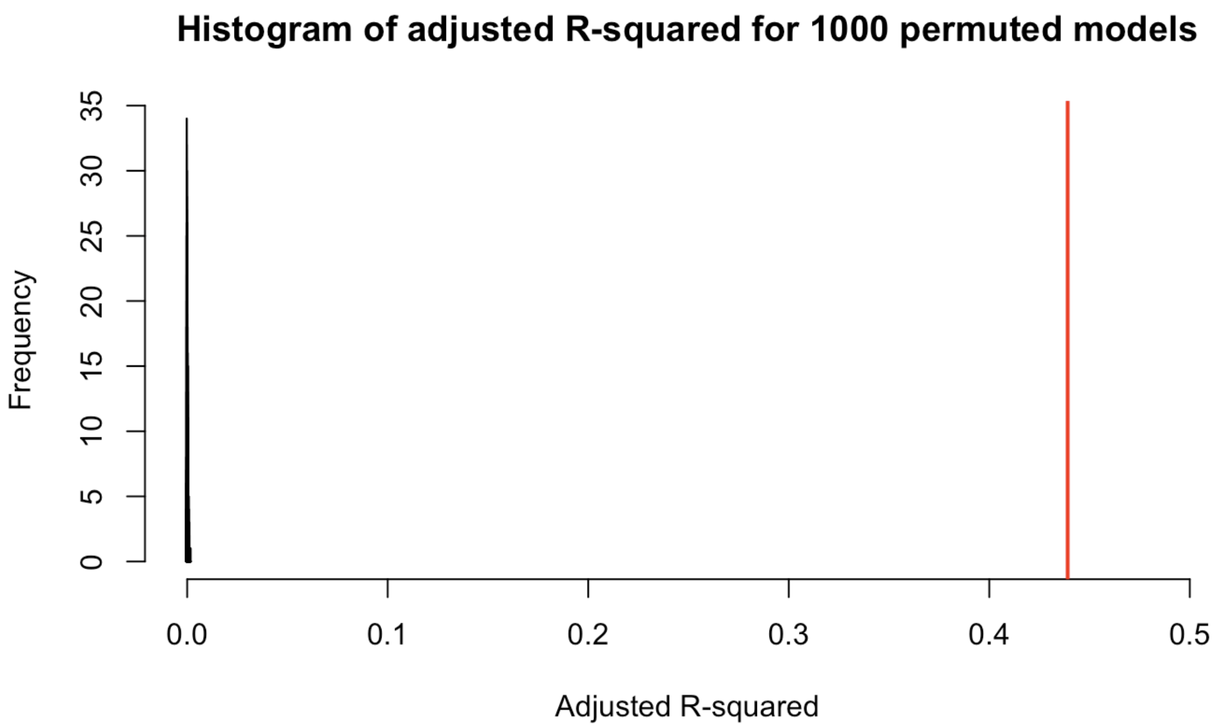

Figure S6: Histogram of adjusted R-squared values for 1000 permuted models. To ensure our linear model does not overfit to the available data, we undertook a sequence of permutation tests varying different parts of the data. We randomly permuted the 9408 values of the dependent variable (49 strains tested across 192 environments) 1000 times and fitted the same linear regression model to each permutation, recording the adjusted R-squared of each model. The exceptionally narrow distribution around zero on the left shows that permuted values could not be explained by our dependent variables. The red line indicates the adjusted R-squared of the original model (i.e., fit to the correctly ordered dependent variable values). None of the resulting models showing any fit to the data.

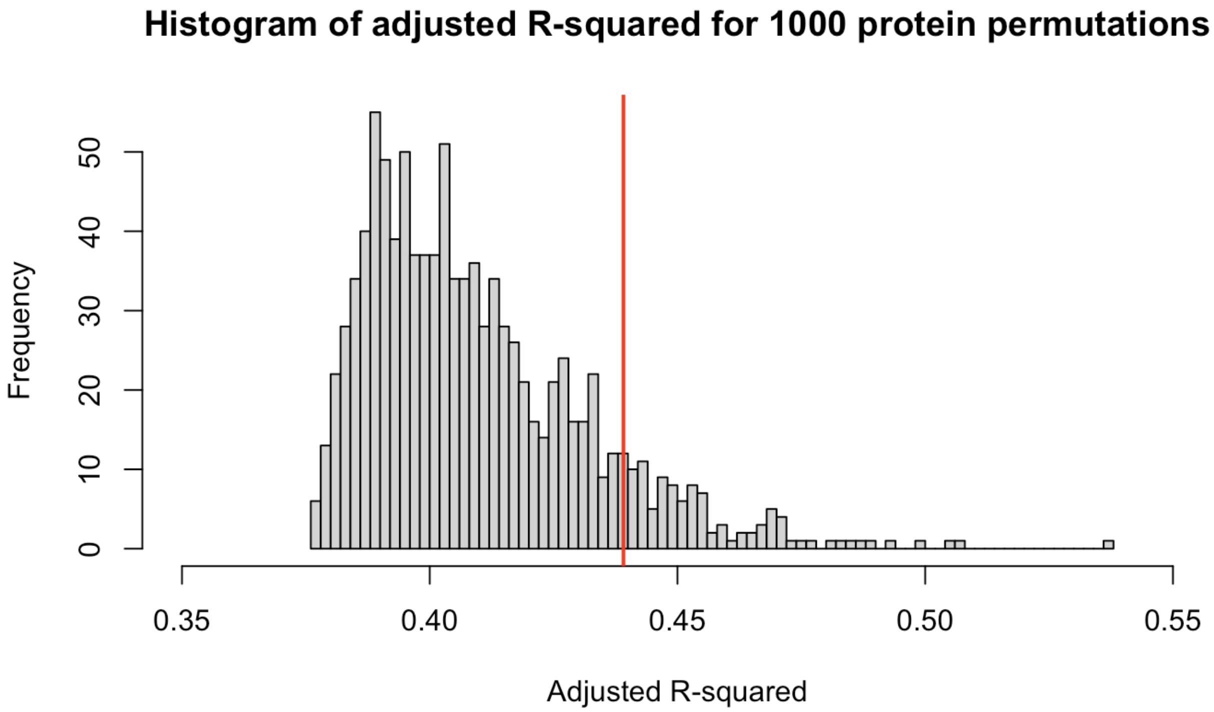

Figure S7: Controlled protein permutation testing. We performed additional permutation testing of the protein information by keeping the environments in the correct order but individually permuting the set of strain/protein values within each environment. All of the resulting strain/protein-permuted models returned adjusted R-squared values of 0.376 (to three significant figures), indicating consistent loss of significance for network features, as intended (that is, when the wrong strain/protein information is provided). Thus, the protein information is indeed statistically significant in explaining around 6% of the amount of biofilm formed for phenotypes. We achieved a larger adjusted R-squared with nonlinear network terms, but some of them do not meet any reasonable p-value threshold; for simplicity we thus restrict our attention to linear models. Keeping the environments correctly ordered, we applied the same random permutation of proteins to all environments per run; that is, the protein information was not completely random, but it was incorrectly ordered in the same way in each environment. The red line indicates the adjusted R-squared of the true model. Notably, the fit to the permuted data provided higher adjusted R-squared than for the true model in 10.3% of these constrained permutations. Whereas such results might at first glance indicate possible overfit, we suspect that the small size of the model allowed for many permutations where the protein types (cf. specific proteins) were mapped closely enough that such results should not be surprising. That is, good results might reasonably occur if high-degree duals were largely mapped onto high-degree duals (about a fourth of the whole network), and low-degree PilZ-binding proteins were mapped onto low-degree PDEs or DGCs, which, at least in terms of their PPI network centralities are practically indistinguishable as groups. Hence, while the network features are statistically significant and are not vulnerable to completely random effects, they do not possess finer resolution that would allow them to differentiate between proteins that behave quite similarly in the PPI network. We interpret the results in here as indicating that the main contribution from the PPI network protein features may be mostly tied to the protein type (dual, PDE, DGC, PilZ) as opposed to the specific protein identities within those types. We also advise carefully permutation testing any nonlinear larger-scale models, as the chase for finely tuned protein information might make a model even more vulnerable to random effects.

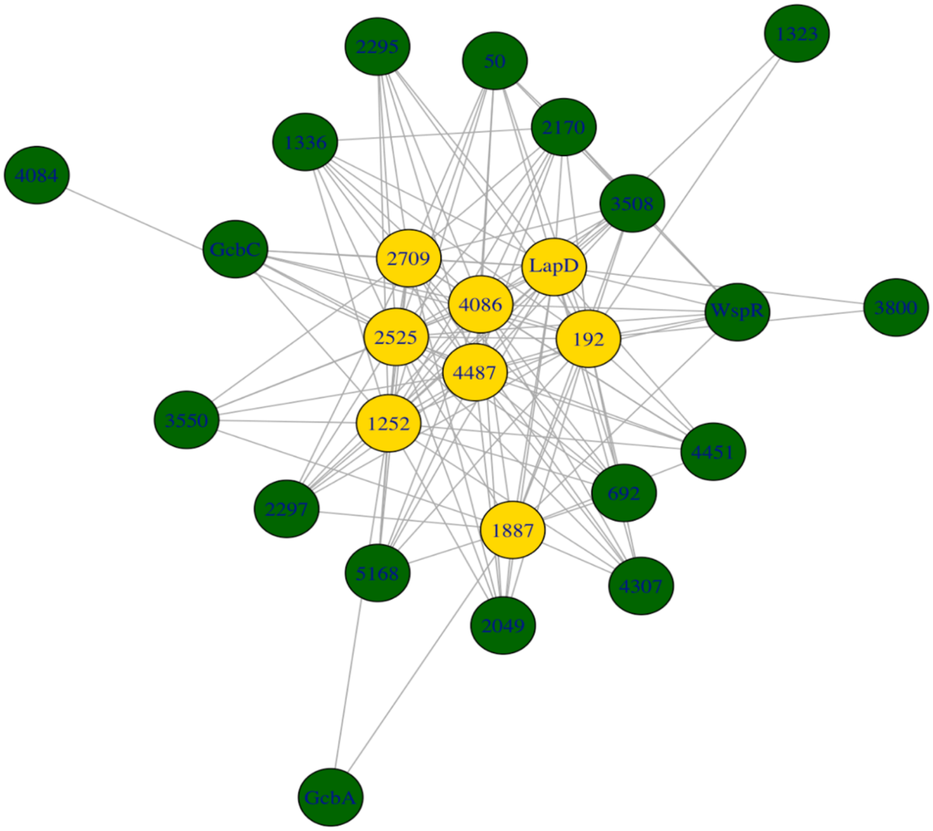

Figure S8: Subgraph of the PPI network as a "Hub Model' visualization. The eight highest degree dual-domain proteins (with degree 17 and above, yellow) and all DGCs (green) that interact with them are included as nodes in the induced subgraph of the PPI network. Only three DGCs were excluded from the subgraph, since they do not interact with these dual-domain proteins: Pfl_0190, GcbB, Pfl_2176. The large graph layout in the igraph package was used for the subgraph visualization. These core dual-domain and DGC interactors play crucial role in the localized signaling of the "Hub Model".
