# Supplementary material for: Local control: a hub-based model for the c-di-GMP network
Source: mSphere. 2024 Apr 9;9(5):e00178-24. doi: 10.1128/msphere.00178-24 (PMC11237430; doi:10.1128/msphere.00178-24)
Supplement: Supplemental Text — Supplemental methods. [file msphere.00178-24-s0002.pdf]

SUPPLEMENTAL METHODS

Network features and PCA

We computed the following network features for each node: degree, betweenness, eigenvector, PageRank, harmonic, and subgraph centralities, local clustering coefficient, local efficiency, and average nearest neighbor degree [1]. Harmonic centrality is a variant of closeness centrality that is well-defined on disconnected networks; its values are highly correlated with closeness centrality scores on a connected network. The network has six singletons (Pfl_190, Pfl_958, Pfl_2176, Pfl_3972, Pfl_4150, Pfl_4257), whose centralities values are trivially zero. Therefore, to avoid singletons skewing the multidimensional scaling of the centralities we performed PCA on the 43 proteins within the largest connected component.

We centered and scaled all network features, as subgraph centrality has disproportionally large values and skews PCA results. Roughly, local clustering coefficient, local efficiency, and average nearest neighbor degree measure how well-connected the neighborhood of a node; betweenness tries to detect if a node acts as a bridge between well-clustered groups; other centralities measure the importance of the node itself via neighbor or shortest path counts, or the spectrum of the adjacency matrix [1].

Logistic regression and protein type classification

Classifiers that used principal component coordinates yielded lower AUC scores than classifiers above with network features as independent variables.

Strain processing and clustering

All strains were grown in thirteen batches in two Biolog plates with a total of 192 carbon sources in base minimal medium, as reported [2]. Wild type was grown as a control in each batch, which allowed us to compute the average wild type biofilm biomass in each of the 192 environments and assess the variance of wild type growth in each batch of assays. To control for day-to-day variation, we normalized each batch by the ratio between median wild type growth in a given batch over the average of medians of wild type growth in all batches. The distribution of data appears to be relatively normal, with median close to mean in all batches; the choice of normalization by median ratios was arbitrary. Then, the eight strains that were tested in more than one batch were averaged before hierarchical clustering on all batches and strains was performed.

Linear models

All network variables were centered and scaled. Amount of biofilm formation in a wild type is on the same scale as that of a phenotype, and thus, was not scaled. Cross-validation was not performed due to small number of observations (only 49 interacting proteins).

We performed a multivariate logistic regression model using eigenvector, betweenness, and PageRank centralities, and with many univariate and other multivariate models to test the prediction strength of each independent variable, with results in **Table S1/S2**. As expected from the visualization in **Figure 1**, the four variables that align most with the first principal component (eigenvector, PageRank, degree and betweenness) are quite powerful in predicting the protein types. PageRank, degree and eigenvector are almost equally good at detecting dual domain

proteins and PDEs in univariate models, which aligns with variation of these types along the first principal component (**Figure 1**). However, the PageRank, degree and eigenvector features do not perform better than random (AUC < 0.5) at classifying DGC proteins, since most of the variation associated with DGC appears to lie along the second principal component (**Figure 1**). For comparison, average nearest neighbor degree, with most of its loadings in PC2 and PC3, more accurately classifies DGCs, but does not perform as well for dual-function protein and PDEs (**Table S1/S2**).

We next look at a combination of these features. The combination of three network topological features, namely, eigenvector, PageRank, and betweenness (**Table S1, red row**), yields the highest classification performance if we take into account model parsimony and statistical significance of input variables.

References.

1. Newman, M. E. J. Networks: An introduction. Oxford: Oxford University Press, 2010.
2. Dahlstrom KM, Collins AJ, Doing G, Taroni JN, Gauvin TJ, Greene CS, Hogan DA, O'Toole GA. A multimodal strategy used by a large c-di-gmp network. J Bacteriol. 2018 Mar 26;200(8):e00703-17. doi: 10.1128/JB.00703-17. PMID: 29311282; PMCID: PMC5869470.
