# Supplementary material for: Local control: a hub-based model for the c-di-GMP network
Source: mSphere. 2024 Apr 9;9(5):e00178-24. doi: 10.1128/msphere.00178-24 (PMC11237430; doi:10.1128/msphere.00178-24)
Supplement: Supplemental Tables — Tables S1 to S3. [file msphere.00178-24-s0003.pdf]

SUPPLEMENTAL TABLES

Table S1: AUC scores of logistic regression models (rows) for binary classification of three protein types (columns) participating in c-di-GMP signaling network. The associated P values for some of the data in this Table are shown in **Table S2**.

	**Dual**	**DGC**	**PDE**
eigenvector	0.7371324	0.4897959	0.6613636
PageRank	0.7738971	0.4642857	0.6704545
degree	0.7564338	0.4727891	0.6636364
betweenness	0.7389706	0.5595238	0.5772727
avg nearest neighbor deg	0.6783088	0.7763605	0.5409091
deg + PR + avg_nn_deg	0.7628676	0.7534014	0.6795455
eigen + PR + btwnnss	0.9246324	0.8979592	0.7431818
deg + PR + btwnnss	0.8400735	0.6904762	0.6750000
deg + eigen + btwnnss	0.8658088	0.8843537	0.7568182
eigen + deg + PR + btwnnss	0.9264706	0.8962585	0.7750000
all five centralities	0.9191176	0.9047619	0.7840909

Table S2: P-values of each network feature in the three binary classifiers of corresponding protein types for select analyses shown in **Table S1**.

	**Dual**	**DGC**	**PDE**
eigenvector	0.009702482	0.005065715	0.366506503
PageRank	0.006520128	0.005662699	0.586409013
betweenness	0.02163542	0.01310859	0.86801627

Table S3: Linear regression model with diffused gene additive log ratios[a].

	**coefficient**	**p-value**
(Intercept)	0.0783049	2.01e-08 (***)
wild type biofilm formation	1.0129670	< 2e-16 (***)
betweenness	0.0001193	0.388
eigenvector centrality	0.0750168	1.10e-08 (***)
gene expression diffused	0.0016159	0.521

[a]In various simple linear regression models, gene transcription data did not show any statistical significance, including the model above: *phenotype ~ wild type + betweenness + eigenvector c-ty + gene data* done in a subset of 26 environments. Note: This subset of 26 environments was chosen at random, inclusion of the whole dataset introduces more noise and does not improve statistical significance of diffused gene data.

.
